# Lived experience and lessons learned from the support of two secondary eye care units to improve cataract surgery and refractive services in two regions of Ethiopia: health system-strengthening support

**DOI:** 10.1093/inthealth/ihae074

**Published:** 2024-11-05

**Authors:** Tsegaye Alemu, Dawit Seyum, Samson Tesfaye, Alemayehu Sisay, Andrew Wardle, Mende Mensa Sorato

**Affiliations:** Department of public Health, School of Public Health, College of Medicine and Health Sciences, Hawassa University, Hawassa, Po Box 193, Ethiopia; Eye Health department, Orbis International Ethiopia, Addis Ababa, PO BOX 23508, Ethiopia; Eye Health department, Orbis International Ethiopia, Addis Ababa, PO BOX 23508, Ethiopia; Eye Health department, Orbis International Ethiopia, Addis Ababa, PO BOX 23508, Ethiopia; Eye Health department, Orbis UK, London, Po Box, 4385, United Kingdom; Department of Pharmacy, School of Medicine, Komar University of Science and Technology, Qularaisi, Sulaimaniyah, KRI, Po Box 144, Iraq

**Keywords:** cataract surgery, Ethiopia, health system strengthening, refraction error service, secondary eye care unit

## Abstract

**Background:**

Blindness and poor eyesight are significant public health issues globally and specifically in Ethiopia. In Ethiopia, there is limited access to cataract surgery and refractive error treatment centers. Therefore, this study aimed to evaluate the role of health system support in improving access to eye care services, particularly cataract surgery and refractive error treatment services in two secondary eye care units (SECUs).

**Methods:**

A parallel mixed-lived experience study was conducted in two SECUs. A desk review of relevant project documents and health facility activity reports was performed for quantitative data. The study was conducted from 12 December 2022 to 30 January 2023. A total of 21 in-depth interview participants were included.

**Results:**

Overall, 14 106 cataract surgeries were performed during the pilot project implementation period. Assela Teaching and Referral Hospital Eye Care Unit performed cataract surgery 20–30 times per week. Similarly, Debre Tabor Comprehensive Hospital performed cataract surgery 18–24 times per week. The refractive service of the two SECUs was planned to range from 15–30 to 60 per week, and the refractive services were increased from 50–60 per week in both eye care units.

**Conclusions:**

This project has significantly helped the existing health system achieve the 2030 global target and has improved access to eye care services in selected SECUs. The use of cataract surgery and refractive error performance markedly increased from 0 to 100%.

## Introduction

At least 2.2 billion individuals worldwide experience near or distance vision impairment. Nearly one-half of these instances, or at least 1 billion people affected by visual impairment, might have been avoided or not addressed. These 1 billion people include those who have cataracts (94 million), untreated refractive error (RE; 88.4 million), moderate to severe distant vision impairment or blindness.^1,2^ According to the Lancet Global Health 2021 report, the leading global causes of blindness in those aged ≥50 y were cataracts, glaucoma followed by undercorrected RE, age-related macular degeneration and diabetic retinopathy.^3^ Therefore, vision loss and blindness continue to cause significant losses in health.^4^

A systematic review and meta-analysis conducted in Ethiopia revealed that the overall prevalence of blindness was 1.18%.^5^ Another systematic review and meta-analysis conducted on the prevalence of visual impairment and associated factors among children in Ethiopia revealed that the pooled prevalence of visual impairment was 7%.^6^ This proportion of avoidable blindness and visual impairment is a public health problem in Ethiopia. Evidence indicates that a large proportion of those affected (90%) live in low- and middle-income countries (LMICs). Of these, >90% of people with vision impairment have a preventable or treatable cause with existing highly cost-effective interventions.^7,8^ Furthermore, vision impairment severely impacts the quality of life among adult populations. Adults with vision impairment often have lower rates of workforce participation and productivity and higher rates of depression and anxiety. In older people, it can contribute to social isolation, difficulties in walking and a greater risk of falls and fractures.^9^ In addition, vision impairment imposes an enormous global financial burden, with an estimated annual global productivity loss of approximately US$411 billion in purchasing power parity.^9,10^ In addition, the cataract surgical rate and economic indicators are closely associated, indicating the strong influence of resource availability on healthcare delivery.^11^

The World Health Assembly (WHA) has endorsed global targets for effective coverage of REs and cataract surgery to be achieved by 2030 (a 40% increase in coverage of REs and a 30% increase in coverage of cataract surgery).^12^ Similarly, in 2020, the government of Ethiopia also endorsed WHA resolution no. 73.4 on Integrated People-centered Eye Care (IPEC), including the resolution of preventable vision impairment and blindness. This resolution commits member states to make eye care an integral part of efforts to achieve universal health coverage (UHC) and implement IPEC as part of its national health system.^13^

In Ethiopia, cataract surgery has only been performed by a few ophthalmologists in the past, and very few surgeries have been performed. Because of this problem, the government trained middle health professionals for cataract surgery (called ‘cataract surgeons’) to increase the cataract surgery rate (CSR), but even with this additional resource deployed, the CSR was very low. Therefore, the government of Ethiopia has recognized blindness as one of the major public health problems of the country and has developed a 5-y eye health strategic document, in which the main strategic focus areas are disease control, human resources for eye health, infrastructure for eye health and partnership.^14^ A tracer study was subsequently conducted by Orbis International Ethiopia (OIE) and the Federal Ministry of Health (MOH) to determine the bottlenecks of cataract surgery. The bottlenecks identified were lack/little technical capacity at existing health facilities, shortages of consumables, supplies and medical equipment, poor leadership and government support, medical equipment maintenance/biomedical engineering issues, lack of on-job training for eye care staff, low service uptake and poor patient awareness.^15–17^ Based on the findings, national stakeholders developed action plans and agreed on the response to address the gap and a mean time-designed project. During project design, the stakeholders mainly focused on WHO health system building blocks, such as leadership and governance, service delivery, health workforces, financing, health information and essential medicine/technology.^18^ Therefore, National Institutional Support (NIS) was planned to be implemented from 2014 to December 2022 to support three secondary eye care units (SECUs) to improve cataract surgery and refractive services in these regions.^19^ Therefore, this study aimed to evaluate the role of health system support in improving access to eye care services, particularly cataract surgery and RE treatment services, in two SECUs.

## Methods and materials

### Study design and study period

A parallel mixed-method study involving a quantitative and qualitative design was used. In addition, case studies were documented at the patient level, and direct observations of project activities on the ground were triangulated. In this parallel mixed study design, the authors integrated methods, and qualitative and quantitative data were collected and analyzed concurrently. However, separate analyses were conducted, then integration was performed by merging. In conclusion, integration occurs at the study design level, method level, data collection, analysis and interpretation and reporting level. This mixed study design aimed to enrich the study's findings. The study was conducted from 12 December 2022 to 30 January 2023.

### Pilot project implementation setting

Debre Tabor Comprehensive Hospital and the Assela Teaching and Referral Hospital Eye Care Unit were included in this study. The South Gondar Zone's capital, Debre Tabor, is located in Ethiopia's Amhara Region. Debre Tabor General Hospital provides services to the city’s population (84 382) and its catchment area in the South Gonder Zone (2 609 823).^20^ Regarding human resources, the hospital has two ophthalmologists, one cataract surgeon and three optometrists. Concerning infrastructure, the hospital has three examination rooms, one minor operation room (OR) and one major OR.^21^ Assela is the capital city of the Arsi zone. The Assela Referral & Teaching Hospital under Arsi University serves approximately 3.2 million people in its catchment area. It serves as a referral hospital for four district hospitals and 23 health centers. The hospital has two ophthalmologists, one cataract surgeons, three ophthalmic nurses and three optometrists. Regarding infrastructure, the hospital has two examination rooms, one separate OR and two inpatient rooms, each with three beds.^22^

### Sampling technique

A purposive sampling method was used to collect quantitative data (of the three total supported SECUs, two were included). A consecutive review of relevant documents during the data collection period was performed to evaluate cataract surgery and refractive eye service performance under an essential support program.

### Sample size determination

All relevant documents that fulfilled the inclusion criteria were reviewed among the selected hospitals. For the qualitative part, 21 key informants who believed that they had rich information concerning eye care service status, challenges and essential pilot support programs for SECUs, were included in the in-depth interviews.

### Inclusion and exclusion criteria

Complete adult patient cards (aged ≥18 y) with a diagnosis of cataract, vision loss, impairment, RE or physician referral to eye care units were included. Patient charts with incomplete information and patients aged <18 y were excluded.

### Data collection tool and procedures

In-depth interviews were used to collect qualitative data by using interview guides. Twenty-one study participants (ophthalmologists, cataract surgeons, ophthalmic nurses, optometrists, other eye care professionals, hospital medical directors, hospital chief executive officers, hospital human resources team leaders, hospital finance team leaders, OIE program directors, OIE program managers and OIE monitoring and evaluation managers) were included in the in-depth interviews. In-depth interviews were recorded, and field notes were taken with consent from each study participant. The recorded interviews were translated and transcribed at the end of each day of research. A patient chart review was conducted using a data abstraction format designed for this purpose.

### Data quality assurance techniques

In this study, the trustworthiness of the qualitative data was maintained through credibility, transferability, debriefing, dependability and other criteria. To ensure study credibility, we first utilized a key informant interview (KII) guide that was reviewed by Orbis International Experts and Hawassa University professors. Before actual data collection began, the instruments (discussion and interview guides) were pretested on three in-depth interview participants among health professionals who had experience with eye health service provision and eye health program management. For the second method, we used peer debriefing, which was carried out by qualitative experts from Hawassa University staff. This methodology primarily focused on the coding and analysis of qualitative data. One day of training was given to the data collectors and supervisors.

### Data analysis

Quantitative data were entered into Epi info for each questionnaire and checklist then exported to SPSS version 27 (IBM Company, TU Berlin). Descriptive analyses were performed (e.g. the proportions of eye care activities, means, SDs, frequency tables and figures). Furthermore, in-depth interview recordings were transcribed and checked for accuracy by two authors. The transcripts were read several times for familiarization with the content and then coded, before debriefing and thematic coding of the transcribed and translated in-depth interviews. Then the data were analyzed and compiled using a thematic approach with the support of Atalsti software.

## Results

### Achievements of the selected SECUs during the project pilot period

**Figure 1.**
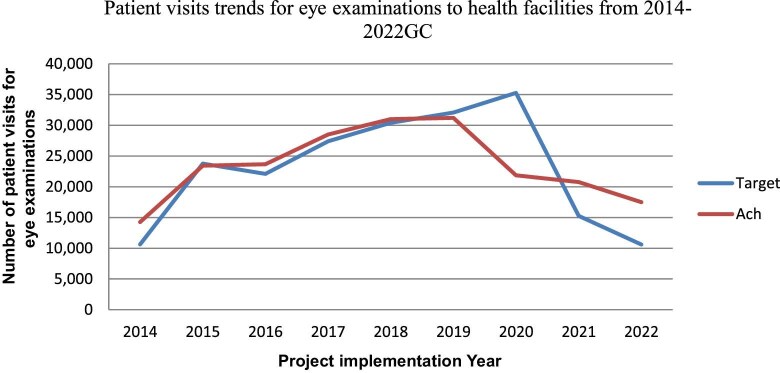
Number of patient visits for eye examinations at three secondary eye care supporting units from 2014 to 2022.

The number of patient visits/flows for eye care examinations at three SECUs during implementation of the pilot project increased substantially (i.e. from 0 to 100% of the target). An increase in patient flow was noted in all project implementation periods except for 2020 (62% [n=21 872]) and 2021. Possible reasons for this could have been the coronavirus disease 2019 (COVID-19) pandemic and the conflict in Tigray (Figure 1).

**Figure 2.**
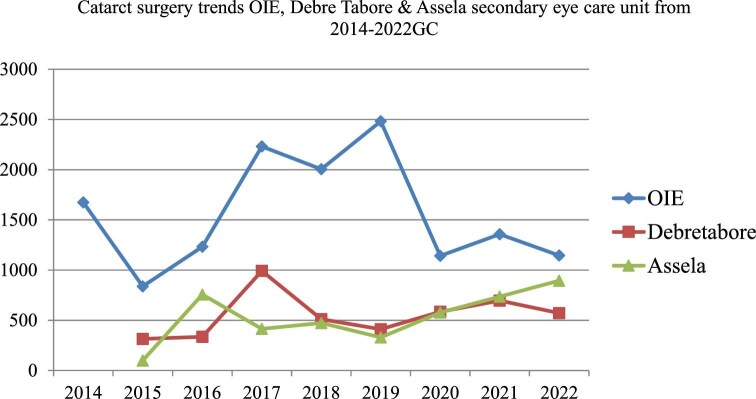
Cataract surgery trends at three secondary eye care supporting units from 2014 to 2022. OIE, Orbis International Ethiopia.

Regarding cataract surgery performance during the project period from 2014 to 2022 at two SECUs, the mean (SD) of cataract surgery performance was 1567.3±561.9, ranging from 838 (2015) to 2482 (2019). Overall, 14 106 cataract surgeries were performed in 9 y. This was a substantially higher accomplishment compared with a previous similar period. The performance of the Debre Tabor SECU in cataract surgery was slightly better than that of the Assela SECU (4418 [Debre Tabor] vs 4282 [Assela Hospital]). There was a slight decrease in cataract surgery performance in 2018 (65%, n=2004) and 2020 (31%, n=1142). The possible reasons for this identified during the in-depth interviews were political instability: the war between the Tigeri region and the Amahara region occurred during the project implementation period and most of the hospital services were shifted to manage emergency patients from war incidents. In addition, the COVID-19 pandemic occurred; because of the pandemic, there were movement restrictions and lockdowns of patients in their homes, which affected patient flow during implementation of the pilot project (Figure 2).

**Figure 3.**
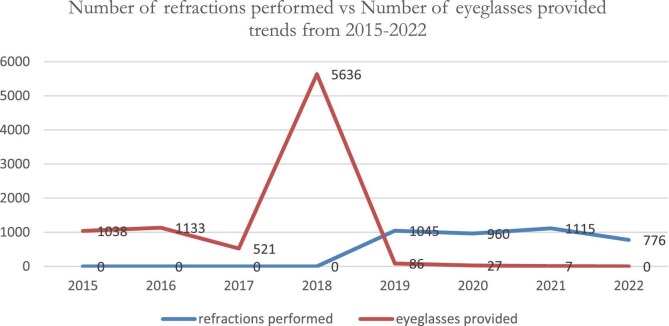
Number of refractions performed vs number of eyeglasses provided trends from 2015 to 2022.

Similarly, refraction services were initiated in 2019. The RE values from 2019 to 2022 were 1045 (16%), 960 (13.3%), 1115 (55.6%) and 776 (78%), respectively. These data indicate that there was progress in refraction service performance (16% vs 78%). In addition, during 2015–2022, 73.8% (n=8448) of patients received eyeglasses, after which the proportion of eyeglasses provided during the given period decreased (Figure 3). RE is the third leading cause of blindness. During project implementation, the hospitals realized that RE was a major issue. In the meantime, there were low-cost technological opportunities; also, MOH-assigned optometrists established optical workshops at Debretabor and Maichewu.

One in-depth interview participant said,

*Before this project was initiated, cataract patients were referred to Adama and Addis Ababa. Due to this, patients stayed for 2–3 days, which created unnecessary costs*.

Another participant noted that:

*There is no doubt about the relevance of the project; there are no things such as restoring visions; you can feel joy for those communities who can gain sight; meanwhile, it is a joy of management and community*.

### Effectiveness and efficiency of the project

The project has shown remarkable progress from the baseline and has achieved its objectives. The Assela SECU plans to increase the number of cataract surgeries from 0 to 8 per week. However, cataract surgical productivity dramatically increased to 20–30 per week. Similarly, the Debre Tabor SECU planned to increase the number of cataract surgeries from 4 to 12 per week but achieved an increase of 18–24 per week.

The project achieved its goal of increasing the refractive services of the three SECUs from 15–30 to 60 per week. Furthermore, surgical capacity significantly increased in terms of the number of people who underwent surgery. In addition to cataract surgery and refractions, the eye care services provided included trauma treatment, conjunctivitis treatment, glaucoma screening and treatment, retinal screening and other treatment services. One in-depth interview participant stated the following:

*Even though our support is decreased every year, the service level increased outcome is achieved. In this pilot project, with minimal support, the secondary eye care unit is continuously functional with all challenges* (40-y-old program manager).

Another participant said:

*The pilot project included a limited budget for equipment and supplies such as an operating microscope, retinoscopy, other equipment, supplies and large volume medication. Most of the procurement was facilitated by OIE from the wholesale market, not the retail market, resulting in significant cost savings* (35-y-old program officer).

Furthermore, project efficiency was demonstrated by the following key practice: initially, the project was assigned a field coordinator at each hospital, but later, a role shift occurred from the field coordinator to the department. This has reduced unit costs without administrative costs. From a health facility eye care services perspective, volume at work has increased without substantial budget allocation, meaning that unit costs decreased over time, but the output either increased or remained constant. An additional element that contributed to project effectiveness was OIE support for full optical workshop equipment and supplies at both Debre Tabor and Machiew Hospital. Optical workshop support significantly increased patient follow-up in both SECUs.

The total budget available was 27.13 million ethiopian birr, and the actual expenditure was 25.51 million ETB, which is 94% of the budget utilization. More money was spent on the actual program (such as equipment, supplies and medication) than on the administration costs. Therefore, the pilot project is significantly more efficient. The research team confirmed that the project is highly efficient. A possible explanation for this could be that the pilot project utilized a dual management system that contributed substantially to efficiency.

#### Institutional development

Concerning institutional capacity building, KIIs and desk review data indicated that there was various capacity-building training for cataract surgeons on manual small incision surgery for 3 wk, that of ophthalmic nurses on Operating Theater, low vision training for optometrists, that ophthalmic equipment maintenance and biomed technicians training was given in India, that travel costs and trainees’ expenses were fully covered by OIE.

Regarding human resource development, before the project, there were few eye care health workers in each SECU. Those available were overworked, undercompensated, unrecognized and very poorly motivated. To help address staff shortages, two nurses, one optometrist, one cataract surgeon and one biomed technician were trained by Debre Tabor SECU. Similarly, three nurses, one cataract surgeon and one biomed technician were trained at the Assela SECU.

Before the project's implementation, the managers of SECUs had no input into the training, compensation or deployment of staff and had limited knowledge or skills for motivating staff. To address these gaps, different types of on-the-job training were given to non-doctor eye healthcare workers, such as ophthalmic nurses, optometrists, cataract surgeons and biomed technicians, through the OIE NIS program.

Furthermore, to provide quality eye care, the project provided equipment such as biometry, A-scan, B-scan, a slit lamp, an operation microscope and consumables and supplies at each SECU.

The human resource capacity building and provision of medical equipment and supplies helped to expand eye care services, establish a resilient eye care service delivery system and enable the establishment of a referral system from primary and tertiary systems (Table S1).

#### Human resources availability

Before the project started, a baseline survey was conducted with eye care professionals working in the partner SECUs. The findings indicated very low staffing: only three eye care professionals in the Debre Tabor and Assela SECUs were involved. During project implementation, eye care professionals were recruited to work in both SECUs (Debre Tabor [10] and Assela [15]). The existing eye care staff were verified via field observation. These factors increase the prevalence of cataracts, REs and other eye care services. Moreover, for those who experience eye care referrals, customer satisfaction has improved. Furthermore, eye care staff retention dramatically improved.

#### Public awareness measures

Public awareness creation through health education can help to prevent blindness and other eye diseases in resource-limited settings. In this project, public awareness creation was conducted in both Debre Tabor and Assela SECUs via routine eye health education to patients and caregivers. Middle-level health professionals, such as ophthalmic nurses, optometrists, health extension workers, schoolteachers and other health professionals, are directly involved in routine eye health education and patient-screening activities.

#### Quality assurance

Quality assurance (QA) is any systematic process of determining whether a service meets specified requirements. QA in this project aimed to monitor activities, strengthen eye service delivery, provide feedback and action points based on the identified gaps, and help to deliver the intended service in the project time. Therefore, the project implemented QA activities such as partner consultative and review meetings, data verification work and quarterly reporting.

#### Secondary eye care infrastructure

When the NIS pilot project started, Debre Tabor and Assela Hospital had one or only a few rooms for eye care service provision, and these were shared with other services. In addition, the services were not given adequate attention from hospital management members or other stakeholders. During the implementation of the NIS project, the OIE carried out many advocacy, promotion and communication activities with high-level officials to allocate dedicated rooms to the SECUs. After this, relatively better dedicated eye care rooms were allocated. However, the rooms are still not adequate, as noted by most of the in-depth interview participants, for example, ‘The services provision space is very limited, narrow, and dispersed, meaning that eye care OPD [outpatient department], inpatient, and OR are located in a different place in Assela’. The researchers observed the infrastructure challenge in both SECUs (Debre Tabor and Assela).

### Key lessons learned from the pilot project implementation

Due to limited capacity, limited resources are available for delivering a large number of eye care services at SECUs, which can help to provide high-level professionals such as ophthalmologists. With minimal support, sustainable and functional eye care service delivery is possible within the existing system (24/7 approach). Resilient elements in eye care systems have developed; most of the existing SECUs were closed during the COVID-19 pandemic, but NIS-supporting SECUs continue their service delivery. In addition to cataract and refractive services, various patient-centered eye care services, such as minor surgery trauma, screening eye care, treating eye disease, and managing and referring complicated eye care, can be offered to tertiary eye care units. In the NIS, sustainable health facilities cannot be developed within 3 y (in and out), and with high volume, each event looks as if it is well known. Encouraging partner engagement is essential for project success, so governmental stakeholders and developmental partner engagements are vital for project success. When the existing healthcare leadership is strong and cooperative, it boosts project performance and becomes better. Therefore, encouraging government ownership and leadership to support the successful implementation of projects is highly advisable; by contrast, when leadership is weak and irresponsible and little attention is given to eye care, it is difficult to achieve the already stated targets. When the NIS project initiated little or no eye care services in two SECUs after technical and supply support, it became a full-blown service for each SECU. The development of human workforces such as on-the-job training significantly increased eye care professionals’ skills and knowledge, improved the quality of service delivery and outreach service delivery, and increased the accessibility of remote and difficult-to-reach communities; these factors helped to address less privileged and demotivated communities.

## Discussion

In this mixed-method study of lived experience, we evaluated the effect of health system-level support provided by a non-governmental organization (NGO) on the public healthcare system in Ethiopia. The project aimed to address challenges in reducing avoidable blindness, such as inequitable access to eye care services, low population knowledge, weak technical expertise and lack of infrastructure capacity of health systems in LMICs.^23^ Support was provided by NIS from 2014 to 2022 to three SECUs (Debre Tabor Hospital, Maichew Hospital and Assela Hospital). Overall, the performance of the SECUs improved significantly with the technical and material support of the OIE. For example, the number of patient visits for eye care examinations at the SECUs increased substantially (i.e. from 0 to 100% of the target). This finding is supported by evidence from the UK showing that creating access to eye care and supporting services for minority communities is critical for addressing their risk of visual impairment.^24^ Another study showed that a clinical decision support system was associated with low vision service utilization.^25^

The mean (±SD) number of patients who underwent cataract surgery was 1567.3 (±561.9), with minimum and maximum values of 838 (2015) and 2482 (2019), respectively. This indicated that the prevalence of more than threefold individuals increased compared to that at baseline, yielding a cataract surgical rate (CSR) of 427 cataract surgeries per million people in 2019. This is higher than findings from a study conducted in southern Ethiopia that showed that the CSR in 2010 was 406 operations per million per year, ranging from 204 to 1349.^15^ This value is also higher than the WHO's expected CSR per year.^11,26–28^ This achievement indicates a promising future for eye care services in general and for cataract surgery if collaborative work based on the equitable distribution of services is undertaken in Ethiopia. This is because the quantity and quality of cataract surgery are enhanced, and more people accessing services is an important indicator of the acceptability and availability of services in the community.^29^ A possible explanation for this achievement could be that SECUs should conducted awareness campaigns and community outreach programs to educate the population about the importance of eye care and early detection of cataracts, and that seeking timely treatment for REs can help increase the use of services in SECUs.

The capacity building of the governmental eye care system is a strategic investment, supporting continuous service provision at the SECUs and increasing the quality and quantity of cataract surgery. Screening and other eye care services were provided to thousands of people, so significant demand was created at the community level. The approach of the project was aligned with guidance from the Vision 2030 targets, in which a 40% increase in RE service, a 30% increase in cataract surgery and screening of at least 80% of people with diabetes for retinopathy can only be achieved through engaging and empowering people, integrating primary care and community-based services with referral linkages, efficient coordinating services, and capacitating and empowering the eye healthcare workforce.^30^ The possible justification could be providing sufficient training and instruction to medical personnel, such as ophthalmologists, optometrists and nurses, to ensure their proficiency in cataract surgical procedures and the management of refractive problems, which are critical for capacity building. Moreover, implementing continuous professional development programs helps to ensure that staff members are kept up to date with the newest innovations in their organization.

Cataract surgery is one of the most cost-effective procedures, and the cost-effectiveness relies on the age at which the condition first manifests itself and the age at which the operation is performed, according to a study that assessed the value of expanding cataract surgery to rural poor people by supporting health systems in low-income countries. When patients are aged ≤78 y, having surgery when serious vision impairment first appears can save money. However, because of a lack of resources, millions of individuals in low-income nations continue to have no opportunity to prevent or treat blindness.^31^

The RE performances from 2019 to 2022 were 1045 (16%), 960 (13.3%), 1115 (55.6%) and 776 (78%), respectively. These data indicate that there was progress in refractive service performance (16% vs 78%). In 2016, 90.4% of refractive people received eyeglasses, a much greater proportion than in other years. Monitoring progress toward effective coverage of eye care services published in the *Lancet* showed that effective cataract surgery coverage and effective RE coverage serve as ideal indicators to track progress in the uptake and quality of eye care services at the global level, as well as to monitor progress towards UHC generally, given the significant unmet need for care associated with cataracts and REs and the existence of highly cost-effective interventions. This finding is supported by a study performed elsewhere on refractive enhancements for residual RE after cataract surgery.^32^

Our study showed that human resource management activities, including on-the-job training, motivation and supportive supervision, also contributed to an increase in the use of cataract surgery and refractive services. This is because eye care requires a sufficient quantity of trained and motivated surgeons (ophthalmologists and cataract surgeons), ophthalmic clinical officers and nurses, optometrists and other eye professionals. The building capacity of human resources for eye care services is one of the core focuses of global action plans to reduce avoidable visual impairment and vision loss.^33^ This is in line with findings in other studies that an adequate number of well-qualified, well-motivated and equitably distributed eye health workers are vital for delivering effective and efficient eye care services. However, there are few eye health workers in most places in LMICs, particularly in sub-Saharan Africa, including Ethiopia. Another study conducted to evaluate the distribution, rates and determinants of cataract surgery service provision in southern Ethiopia also showed that poor staff motivation, community awareness and limited government support were major challenges for cataract surgery performance.^15^ A possible justification could be that on-the-job training for newly employed staff enhances staff capacity to contribute more productivity to cataract surgery and RE.

Improved access to equipment, supplies and infrastructure for eye care services and the integration of tasks into the healthcare system changed the SECUs from performing almost no cataract surgeries to referral centers for eye services. This is supported by evidence from a health system-strengthening approach study that examined the integration of eye health into primary care in sub-Saharan Africa and showed interventions to strengthen human resources at all levels, partnerships and community participation.^34^ A health system dynamics analysis of eye care services in Trinidad and Tobago suggested investments that might reduce avoidable blindness, including more ophthalmic equipment and maintenance in the public sector and pathways, to ensure timely and equitable access to subspecialized surgery. The NIS project strengthened both of these elements (i.e. equipment and referrals). The Trinidad and Tobago study suggested other investments that a continuing NIS project could consider, including screening programs for diabetic retinopathy, retinopathy of prematurity and neonatal eye defects.^35^ The justification could be furnishing the SECU with the required infrastructure, including fully equipped operating theatres, diagnostic equipment for evaluating patients before surgery and facilities aftercare. This is vital for ensuring access to eye care, providing high-quality services in cataract surgery and correcting REs.

An important lesson from this project is that scaling up eye care coverage through collaboration between the government and an NGO can reduce blindness and improve eye health for people in LMICs, particularly because the project improved equitable access to services by focusing on service delivery in rural areas. A study published during the NIS project period supported this focus on equity; due to significant inequalities in access to eye care services, visual impairment is greater in LMICs and underserved populations, such as people living in rural areas, those with low incomes, women, older people, indigenous populations and ethnic minorities.^36^ To ensure the ‘right to sight’, it is clear that new strategies are needed to address current and projected eye care needs in LMICs such as Ethiopia. The NIS project is an example of one such strategy involving local and national partnerships that address health system bottlenecks to ensure equitable access to quality eye care services. The justification could be the accessibility of eye health services for older people, remote communities and disadvantaged communities, which may help to ensure equity^37,38^ and improve access to eye care and may lead to the prevention of avoidable blindness.^39^ Evidence from Rwanda shows that a key learning from the evaluation is the importance of strengthening the eye healthcare system, together with a strong focus on training primary care nurses using a primary eye care curriculum,^40^ while other case studies from Nigeria have indicated that researchers have learned from their experiences in managing these programs and hopefully will provide us with suggestions to improve management.^41^

Our study has policy implications; thus, engaging government and development partners with common goals can inform policy decisions related to eye care services in LMICs.^42^ Therefore, strong and cooperative healthcare leadership boosts project performance. Therefore, it is vital to encourage government ownership and leadership to support the implementation of projects.^43,44^ Similarly, the development of the workforce, for example, with on-the-job training, significantly increased eye care professionals' skills and knowledge, resulting in improved quality of services.^45–47^ In addition, outreach made services accessible for remote, hard-to-reach communities.

Despite its limitations, the findings of this study should be applied. However, the inclusion of two teaching and referral hospital eye care units from only two regions in the country may limit the extrapolation of these findings to other regions.

## Conclusions

Blindness and poor eyesight are significant public health issues in developing countries, particularly in Ethiopia. Therefore, this pilot project was designed to address this gap through health system support in the context of enhancing cataract surgery and RE treatment services. We evaluated the role of health system support in improving access to eye care services, particularly cataract surgery and RE treatment services, at two SECUs. Therefore, the NIS project will help the health system in two SECUs achieve the 2030 global targets of cataract surgery rate and refraction error service. Thus, project scale-up is key to achieving cataract surgery and RE targets. Finally, for researchers willing to study opportunities for avoiding cases of blindness and eye healthcare challenges, it is important to look into local and national potentials that can enhance the technical and financial efficiency of health systems.

## Supplementary Material

ihae074_Supplemental_File

## Data Availability

All data collected during the study are included in this manuscript and the Supplementary Files.
