# Supplementary material for: Lived experience and lessons learned from the support of two secondary eye care units to improve cataract surgery and refractive services in two regions of Ethiopia: health system-strengthening support
Source: Int Health. 2024 Nov 5;17(4):431–9. doi: 10.1093/inthealth/ihae074 (PMC12212216; doi:10.1093/inthealth/ihae074)
Supplement: ihae074_Supplemental_File [file ihae074_supplemental_file.docx]

Table S 1: institutional development characteristics at the debre tabore and assela secondary eye care units

| SN | Activities | Debre Tabor | Assela |
| --- | --- | --- | --- |
|  |  | Performed, Yes/No | Performed, Yes/No |
| 1 | Conduct skill enhancing Hospital Based Program training on Manual Small Incision Cataract Surgery and Operating Theater management | Yes | Yes |
| 2 | Management of operation theatre for Operating Theater nurses | Yes | Yes |
| 3 | Low vision training for optometrists | Yes | No |
| 4 | Ophthalmic equipment maintenance and management training for biomedical technicians | Yes | Yes |
| 5 | Health professionals and teachers training on case identification | Yes | Yes |
| 6 | Documentation and recording of referral cases to and from the secondary eye care units | No | Yes |
| 7 | Provision of equipment including Biometry and supplies | Yes | Yes |
| 8 | Retain trained staff by incentivizing ophthalmic staff | No | Yes |
| 9 | Renovation of availed outpatient, inpatient and Operating Theater rooms | Yes | No |
| 10 | Procure and install additional essential medical equipment | Yes | Yes |
| 11 | Procure and supply essential medical supplies | Yes | Yes |
